# 2-{5,5-Dimethyl-3-[2-(pyridin-2-yl)ethen­yl]cyclo­hex-2-enyl­idene}propane­dinitrile

**DOI:** 10.1107/S1600536811001486

**Published:** 2011-01-22

**Authors:** Liuqing Chen

**Affiliations:** a The College of Materials Science and Engineering, Taiyuan University of Technology, Taiyuan 030024, People’s Republic of China

## Abstract

The mol­ecule of the title compound, C_18_H_17_N_3_, with the exception of the –C(CH_3_)_2_ group, is nearly planar [maximum deviation: 0.208 (4), r.m.s. deviation 0.099 (6) Å] and the disubstituted C atom is displaced by 0.679 (2) Å from the mean plane through the remaining non-H atoms. In the crystal, the packing is stabilized by weak C—H⋯π inter­actions.

## Related literature

For the synthesis, see: Lemke (1970 ▶). For a related structure, see: Kolev *et al.* (2001 ▶). For puckering parameters, see: Cremer & Pople (1975 ▶).
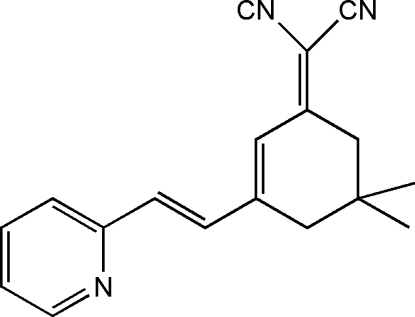


## Experimental

### 

#### Crystal data

C_18_H_17_N_3_
*M* *_r_* = 275.35
Triclinic, 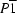

*a* = 8.4910 (17) Å
*b* = 9.6516 (19) Å
*c* = 9.6532 (19) Å
α = 89.06 (3)°
β = 70.47 (3)°
γ = 87.02 (3)°
*V* = 744.6 (3) Å^3^
*Z* = 2
Mo *K*α radiation
μ = 0.07 mm^−1^
*T* = 293 K
0.24 × 0.20 × 0.10 mm

#### Data collection

Bruker SMART APEX CCD area-detector diffractometer
Absorption correction: multi-scan (*SADABS*; Sheldrick, 2008 ▶) *T*_min_ = 0.982, *T*_max_ = 0.993
5037 measured reflections
2617 independent reflections
2076 reflections with *I* > 2σ(*I*)
*R*_int_ = 0.020

#### Refinement

*R*[*F* ^2^ > 2σ(*F* ^2^)] = 0.040
*wR*(*F* ^2^) = 0.112
*S* = 1.07
2617 reflections
192 parameters
H-atom parameters constrained
Δρ_max_ = 0.23 e Å^−3^
Δρ_min_ = −0.20 e Å^−3^

### 

Data collection: *SMART* (Bruker, 2000 ▶); cell refinement: *SAINT-Plus* (Bruker, 2000 ▶); data reduction: *SAINT-Plus*; program(s) used to solve structure: *SHELXS97* (Sheldrick, 2008 ▶); program(s) used to refine structure: *SHELXL97* (Sheldrick, 2008 ▶); molecular graphics: *ORTEPIII* (Burnett & Johnson, 1996 ▶) and *ORTEP-3 for Windows* (Farrugia, 1997 ▶); software used to prepare material for publication: *SHELXTL/PC* (Sheldrick, 2008 ▶) and *PLATON* (Spek, 2009 ▶).

## Supplementary Material

Crystal structure: contains datablocks I, global. DOI: 10.1107/S1600536811001486/dn2652sup1.cif

Structure factors: contains datablocks I. DOI: 10.1107/S1600536811001486/dn2652Isup2.hkl

Additional supplementary materials:  crystallographic information; 3D view; checkCIF report

## Figures and Tables

**Table 1:** Hydrogen-bond geometry (Å, °) *Cg*1 is the centroid of the pyridine ring.

*D*—H⋯*A*	*D*—H	H⋯*A*	*D*⋯*A*	*D*—H⋯*A*
C9—H9*A*⋯*Cg*1^i^	0.97	2.77	3.6933 (16)	160

Symmetry code: (i) 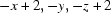.

**Fig. 1.**
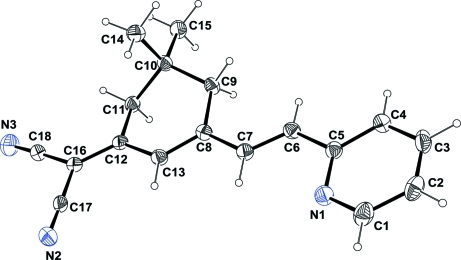
Molecular view of the title compound with the atom labeling for non-H atoms. Displacement ellipsoids are darwn at the 50% probability level. H atoms are represented as small spheres of arbitrary radii.

## supplementary crystallographic information

### Comment

Since discovery of their solvatochromic behaviour (Lemke, 1970), derivatives of 2-(5,5-dimethyl-3-styrylcyclohexenylidene)malononitrile have attracted considerable interest for numerous applications, such as candidates for non-linear optical (NLO), organic light emitting diodes(OLED). As part of our investigations on organic electrooptical materials, 2-(3-(2-vinyl pyridine)-5,5-dimethylcyclohex-2-enylidene)malononitrile (VPDEM)(I) was synthesized according to the general procedure described by Lemke (1970). An X-ray crystal structure determination of (I) was undertaken in order to elucidate the conformation, and the results are presented here.

With the exception of the C(CH_3_)_2_ group, the molecule of the title compound is nearly planar; the disubstituted C atom being displaced by -0.679 (2) Å from the mean plane of the remaining non-H atoms (Fig. 1). The disubstuted cyclohexene ring has an envelope conformation with puckering parameters: Q= 0.4657 (13) Å, θ= 126.97 (16)° and φ= 323.0 (2)° (Cremer & Pople, 1975).The the 2-vinylpyridine is planar with the largest deviation from the plane being -0.0876 (8)Å at C8. The bond distances and angles within the 5,5-dimethylcyclohex-2-enylidene) malononitrile are in agreement with the related 2-(3-(2-(4-Hydroxyphenyl)vinyl)-5,5-dimethylcyclohex-2-en-1-ylidene)-malononitrile (Kolev *et al.*, 2001).

In the crystal, the packing is stabilized by weak C-H···π between the C9 methylene group of the cyclohexene ring and the symmetry related pyridine ring and Van der Waals forces.

### Experimental

The compound was synthesized in a manner similar to the general procedure described by Lemke (1970). And the preparation of compound 2-(3,5,5,-trimethylcyclohex-2-enylidene)malononitrile was previously reported by Kolev (Kolev,*et al.* 2001). Malononitrile (1.87 g, 28.3 mmol) and isophorone (3.90 g, 28.3 mmol) were added to a solution of acetic acid (28µl), acetic anhydride (18µl), piperidine (380µl) and DMF (5.0 ml). The mixture was stirred at room temperature for 1 h and then at 80°C for 1 h. Then pyridine-2-carboxaldehyde (3.3789 g, 0.0122 mol) was added, and the reaction mixture was stirred at 80°C for 1 h. The mixture was poured into 200 ml of hot water containing 6 ml concentrated HCl and the precipitate was washed by water for three times. The solid was collected by filtration under reduced pressure and the crystals were grown from an CH_3_CN solution by slow evaporation at room temperature over a period of several days with a yield of 63%; ^1^H NMR(300 MHz, CDCl_3_): 1.03(s, 6H), 2.57(s, 2H), 2.64(s, 2H), 6.95(s,1*H*), 7.28(d, 2H), 7.64(d, 2H), 7.81~7.87(m, 1H), 8.61(d, 1H); IR(KBr, cm^-1^) ν:3404, 2962, 2224, 1609, 1574, 1522, 1462, 1322, 1262, 1210, 1158, 1097, 960, 890, 760; Anal. Calcd. For C_18_ H_17_ N_3_: C 78.45; H 6.17; N 15.25; Found: C78.41; H 6.17; N 15.22.

### Refinement

All H atoms attached to C atoms were fixed geometrically and treated as riding on their parent atoms with C—H = 0.96 Å (methyl), 0.97 Å (methylene) and 0.93 Å (aromatic) with U_iso_(H) = 1.2U_eq_(C methylene and aromatic) or U_iso_(H) = 1.5U_eq_(C methyl).

### Crystal data

**Table:**

C_18_H_17_N_3_	*Z* = 2
*M**_r_* = 275.35	*F*(000) = 292
Triclinic, *P*1	*D*_x_ = 1.228 Mg m^−^^3^
Hall symbol: -P 1	Mo *K*α radiation, λ = 0.71073 Å
*a* = 8.4910 (17) Å	Cell parameters from 3453 reflections
*b* = 9.6516 (19) Å	θ = 2.5–27.2°
*c* = 9.6532 (19) Å	µ = 0.07 mm^−^^1^
α = 89.06 (3)°	*T* = 293 K
β = 70.47 (3)°	Block, colorless
γ = 87.02 (3)°	0.24 × 0.20 × 0.10 mm
*V* = 744.6 (3) Å^3^	

### Data collection

**Table:**

Bruker SMART APEX CCD area-detector diffractometer	2617 independent reflections
Radiation source: fine-focus sealed tube	2076 reflections with *I* > 2σ(*I*)
graphite	*R*_int_ = 0.020
φ and ω scans	θ_max_ = 25.0°, θ_min_ = 2.1°
Absorption correction: multi-scan (*SADABS*; Sheldrick, 2008)	*h* = −10→10
*T*_min_ = 0.982, *T*_max_ = 0.993	*k* = −11→11
5037 measured reflections	*l* = −8→11

### Refinement

**Table:**

Refinement on *F*^2^	Primary atom site location: structure-invariant direct methods
Least-squares matrix: full	Secondary atom site location: difference Fourier map
*R*[*F*^2^ > 2σ(*F*^2^)] = 0.040	Hydrogen site location: inferred from neighbouring sites
*wR*(*F*^2^) = 0.112	H-atom parameters constrained
*S* = 1.07	*w* = 1/[σ^2^(*F*_o_^2^) + (0.0694*P*)^2^] where *P* = (*F*_o_^2^ + 2*F*_c_^2^)/3
2617 reflections	(Δ/σ)_max_ < 0.001
192 parameters	Δρ_max_ = 0.23 e Å^−^^3^
0 restraints	Δρ_min_ = −0.20 e Å^−^^3^

### Special details

**Table:**

Geometry. All esds (except the esd in the dihedral angle between two l.s. planes) are estimated using the full covariance matrix. The cell esds are taken into account individually in the estimation of esds in distances, angles and torsion angles; correlations between esds in cell parameters are only used when they are defined by crystal symmetry. An approximate (isotropic) treatment of cell esds is used for estimating esds involving l.s. planes.
Refinement. Refinement of *F*^2^ against ALL reflections. The weighted *R*-factor *wR* and goodness of fit *S* are based on *F*^2^, conventional *R*-factors *R* are based on *F*, with *F* set to zero for negative *F*^2^. The threshold expression of *F*^2^ > σ(*F*^2^) is used only for calculating *R*-factors(gt) *etc*. and is not relevant to the choice of reflections for refinement. *R*-factors based on *F*^2^ are statistically about twice as large as those based on *F*, and *R*- factors based on ALL data will be even larger.

### Fractional atomic coordinates and isotropic or equivalent isotropic displacement parameters (Å^2^)

**Table:**

	*x*	*y*	*z*	*U*_iso_*/*U*_eq_	
N1	1.27970 (12)	−0.18230 (11)	0.77938 (12)	0.0255 (3)	
N2	1.19520 (13)	0.35307 (12)	0.25775 (12)	0.0284 (3)	
N3	0.71256 (14)	0.57738 (13)	0.39307 (14)	0.0374 (3)	
C1	1.39339 (16)	−0.27762 (14)	0.79280 (15)	0.0301 (4)	
H1	1.5036	−0.2707	0.7316	0.036*	
C2	1.35746 (17)	−0.38617 (14)	0.89219 (15)	0.0291 (3)	
H2	1.4413	−0.4502	0.8967	0.035*	
C3	1.19582 (17)	−0.39740 (14)	0.98393 (15)	0.0289 (3)	
H3	1.1678	−0.4690	1.0521	0.035*	
C4	1.07473 (16)	−0.29945 (14)	0.97293 (14)	0.0245 (3)	
H4	0.9644	−0.3042	1.0345	0.029*	
C5	1.12001 (14)	−0.19426 (13)	0.86906 (13)	0.0196 (3)	
C6	0.99405 (14)	−0.09422 (13)	0.84861 (13)	0.0197 (3)	
H6	0.8844	−0.0997	0.9117	0.024*	
C7	1.02591 (14)	0.00468 (13)	0.74519 (13)	0.0195 (3)	
H7	1.1372	0.0131	0.6873	0.023*	
C8	0.90367 (14)	0.09977 (13)	0.71496 (13)	0.0182 (3)	
C9	0.72124 (14)	0.09492 (13)	0.80722 (13)	0.0196 (3)	
H9A	0.7055	0.1347	0.9028	0.024*	
H9B	0.6922	−0.0014	0.8223	0.024*	
C10	0.60033 (14)	0.17193 (13)	0.74062 (13)	0.0194 (3)	
C11	0.67159 (14)	0.31267 (13)	0.68002 (13)	0.0199 (3)	
H11A	0.5989	0.3598	0.6333	0.024*	
H11B	0.6737	0.3702	0.7608	0.024*	
C12	0.84488 (14)	0.29522 (13)	0.57089 (13)	0.0184 (3)	
C13	0.95597 (14)	0.19220 (13)	0.60334 (13)	0.0199 (3)	
H13	1.0684	0.1887	0.5455	0.024*	
C14	0.57912 (15)	0.08771 (14)	0.61607 (14)	0.0267 (3)	
H14A	0.5279	0.0026	0.6546	0.040*	
H14B	0.6867	0.0673	0.5435	0.040*	
H14C	0.5094	0.1403	0.5717	0.040*	
C15	0.42935 (15)	0.19588 (14)	0.85935 (14)	0.0260 (3)	
H15A	0.3547	0.2455	0.8181	0.039*	
H15B	0.4412	0.2491	0.9382	0.039*	
H15C	0.3848	0.1081	0.8964	0.039*	
C16	0.89702 (14)	0.37719 (13)	0.44842 (13)	0.0191 (3)	
C17	1.06329 (15)	0.36272 (12)	0.34315 (13)	0.0207 (3)	
C18	0.79250 (15)	0.48667 (14)	0.41784 (14)	0.0234 (3)	

### Atomic displacement parameters (Å^2^)

**Table:**

	*U*^11^	*U*^22^	*U*^33^	*U*^12^	*U*^13^	*U*^23^
N1	0.0191 (5)	0.0285 (7)	0.0261 (6)	0.0033 (5)	−0.0046 (5)	0.0038 (5)
N2	0.0253 (6)	0.0333 (7)	0.0253 (6)	−0.0041 (5)	−0.0067 (5)	0.0063 (5)
N3	0.0384 (7)	0.0344 (8)	0.0440 (8)	−0.0003 (6)	−0.0207 (6)	0.0118 (6)
C1	0.0261 (7)	0.0339 (8)	0.0295 (8)	0.0080 (6)	−0.0096 (6)	0.0005 (7)
C2	0.0344 (8)	0.0250 (8)	0.0327 (8)	0.0088 (6)	−0.0190 (7)	−0.0039 (6)
C3	0.0424 (8)	0.0199 (7)	0.0304 (8)	−0.0028 (6)	−0.0201 (7)	0.0067 (6)
C4	0.0263 (7)	0.0245 (8)	0.0240 (7)	−0.0045 (6)	−0.0098 (6)	0.0043 (6)
C5	0.0217 (7)	0.0190 (7)	0.0191 (6)	−0.0012 (5)	−0.0082 (5)	−0.0004 (5)
C6	0.0172 (6)	0.0211 (7)	0.0189 (6)	−0.0006 (5)	−0.0035 (5)	−0.0002 (5)
C7	0.0169 (6)	0.0209 (7)	0.0187 (6)	0.0011 (5)	−0.0036 (5)	0.0000 (5)
C8	0.0188 (6)	0.0185 (7)	0.0161 (6)	−0.0012 (5)	−0.0042 (5)	−0.0021 (5)
C9	0.0189 (6)	0.0198 (7)	0.0173 (6)	−0.0001 (5)	−0.0026 (5)	0.0023 (5)
C10	0.0172 (6)	0.0215 (7)	0.0179 (6)	−0.0017 (5)	−0.0039 (5)	0.0026 (5)
C11	0.0184 (6)	0.0207 (7)	0.0208 (6)	0.0014 (5)	−0.0072 (5)	0.0015 (5)
C12	0.0195 (6)	0.0177 (7)	0.0199 (6)	−0.0035 (5)	−0.0087 (5)	−0.0003 (5)
C13	0.0159 (6)	0.0216 (7)	0.0204 (6)	0.0000 (5)	−0.0040 (5)	0.0010 (5)
C14	0.0266 (7)	0.0306 (8)	0.0239 (7)	−0.0076 (6)	−0.0089 (6)	0.0013 (6)
C15	0.0190 (7)	0.0321 (8)	0.0245 (7)	0.0016 (6)	−0.0049 (6)	0.0055 (6)
C16	0.0185 (6)	0.0196 (7)	0.0204 (7)	−0.0033 (5)	−0.0077 (5)	0.0018 (5)
C17	0.0258 (7)	0.0187 (7)	0.0213 (7)	−0.0050 (5)	−0.0124 (6)	0.0056 (5)
C18	0.0243 (7)	0.0256 (8)	0.0219 (7)	−0.0058 (6)	−0.0095 (6)	0.0053 (6)

### Geometric parameters (Å, °)

**Table:**

N1—C1	1.3352 (17)	C9—H9A	0.9700
N1—C5	1.3526 (16)	C9—H9B	0.9700
N2—C17	1.1476 (16)	C10—C14	1.5267 (17)
N3—C18	1.1507 (17)	C10—C15	1.5284 (17)
C1—C2	1.384 (2)	C10—C11	1.5415 (18)
C1—H1	0.9300	C11—C12	1.4990 (17)
C2—C3	1.3709 (19)	C11—H11A	0.9700
C2—H2	0.9300	C11—H11B	0.9700
C3—C4	1.3895 (18)	C12—C16	1.3694 (18)
C3—H3	0.9300	C12—C13	1.4374 (17)
C4—C5	1.3904 (19)	C13—H13	0.9300
C4—H4	0.9300	C14—H14A	0.9600
C5—C6	1.4638 (17)	C14—H14B	0.9600
C6—C7	1.3402 (19)	C14—H14C	0.9600
C6—H6	0.9300	C15—H15A	0.9600
C7—C8	1.4490 (17)	C15—H15B	0.9600
C7—H7	0.9300	C15—H15C	0.9600
C8—C13	1.3578 (18)	C16—C18	1.4349 (18)
C8—C9	1.5095 (16)	C16—C17	1.4388 (17)
C9—C10	1.5382 (17)		
			
C1—N1—C5	117.15 (12)	C15—C10—C9	109.57 (10)
N1—C1—C2	124.07 (13)	C14—C10—C11	109.23 (10)
N1—C1—H1	118.0	C15—C10—C11	109.62 (10)
C2—C1—H1	118.0	C9—C10—C11	108.81 (10)
C3—C2—C1	118.69 (12)	C12—C11—C10	111.68 (10)
C3—C2—H2	120.7	C12—C11—H11A	109.3
C1—C2—H2	120.7	C10—C11—H11A	109.3
C2—C3—C4	118.58 (13)	C12—C11—H11B	109.3
C2—C3—H3	120.7	C10—C11—H11B	109.3
C4—C3—H3	120.7	H11A—C11—H11B	107.9
C3—C4—C5	119.43 (12)	C16—C12—C13	121.43 (11)
C3—C4—H4	120.3	C16—C12—C11	121.50 (11)
C5—C4—H4	120.3	C13—C12—C11	117.03 (11)
N1—C5—C4	122.06 (12)	C8—C13—C12	122.75 (11)
N1—C5—C6	117.04 (12)	C8—C13—H13	118.6
C4—C5—C6	120.86 (11)	C12—C13—H13	118.6
C7—C6—C5	124.47 (11)	C10—C14—H14A	109.5
C7—C6—H6	117.8	C10—C14—H14B	109.5
C5—C6—H6	117.8	H14A—C14—H14B	109.5
C6—C7—C8	126.26 (11)	C10—C14—H14C	109.5
C6—C7—H7	116.9	H14A—C14—H14C	109.5
C8—C7—H7	116.9	H14B—C14—H14C	109.5
C13—C8—C7	119.05 (11)	C10—C15—H15A	109.5
C13—C8—C9	121.08 (11)	C10—C15—H15B	109.5
C7—C8—C9	119.87 (11)	H15A—C15—H15B	109.5
C8—C9—C10	114.54 (10)	C10—C15—H15C	109.5
C8—C9—H9A	108.6	H15A—C15—H15C	109.5
C10—C9—H9A	108.6	H15B—C15—H15C	109.5
C8—C9—H9B	108.6	C12—C16—C18	122.74 (11)
C10—C9—H9B	108.6	C12—C16—C17	122.27 (11)
H9A—C9—H9B	107.6	C18—C16—C17	114.97 (11)
C14—C10—C15	108.76 (10)	N2—C17—C16	178.78 (14)
C14—C10—C9	110.84 (10)	N3—C18—C16	177.81 (14)
			
C13—C8—C9—C10	15.38 (17)	C10—C11—C12—C13	−40.03 (15)
C7—C8—C9—C10	−164.63 (10)	C7—C8—C13—C12	−176.48 (10)
C8—C9—C10—C14	76.11 (13)	C9—C8—C13—C12	3.51 (19)
C8—C9—C10—C15	−163.85 (10)	C11—C12—C13—C8	9.42 (18)
C8—C9—C10—C11	−44.02 (14)	C11—C12—C16—C18	1.62 (19)
C14—C10—C11—C12	−65.09 (13)	C13—C12—C16—C17	1.87 (18)
C9—C10—C11—C12	56.03 (13)	C11—C12—C16—C17	179.46 (11)
C10—C11—C12—C16	142.28 (12)		

### Hydrogen-bond geometry (Å, °)

**Table:**

Cg1 is the centroid of the pyridine ring.

**Table:**

*D*—H···*A*	*D*—H	H···*A*	*D*···*A*	*D*—H···*A*
C9—H9A···Cg1^i^	0.97	2.77	3.6933 (16)	160

Symmetry codes: (i) −*x*+2, −*y*, −*z*+2.

## References

[bb1] Bruker (2000). *SMART* and *SAINT-Plus* Bruker AXS Inc., Madison, Wisconsin, USA.

[bb2] Burnett, M. N. & Johnson, C. K. (1996). *ORTEPIII* Report ORNL-6895. Oak Ridge National Laboratory, Tennessee, USA.

[bb3] Cremer, D. & Pople, J. A. (1975). *J. Am. Chem. Soc.* **97**, 1354–1358.

[bb4] Farrugia, L. J. (1997). *J. Appl. Cryst.* **30**, 565.

[bb5] Kolev, T., Glavcheva, Z., Yancheva, D., Schürmann, M., Kleb, D.-C., Preut, H. & Bleckmann, P. (2001). *Acta Cryst.* E**57**, o561–o562.

[bb6] Lemke, R. (1970). *Chem. Ber.* **103**, 1894–1898.

[bb7] Sheldrick, G. M. (2008). *Acta Cryst.* A**64**, 112–122. 10.1107/S0108767307043930 18156677

[bb8] Spek, A. L. (2009). *Acta Cryst.* D**65**, 148–155. 10.1107/S090744490804362X PMC2631630 19171970

